# Correlation analysis between eight anthropometric indicators and arterial stiffness in relatively healthy Chinese adults

**DOI:** 10.3389/fnut.2026.1873108

**Published:** 2026-08-17

**Authors:** Yuting Duan, Hui Lu, Qun Liu, Jie Zhang, Quxinru Wang, Xingzi Zhang, Wenjie Jiang, Wenjun Li, Yiming Chu, Song Hu, Zhe Feng, Weiguang Zhang

**Affiliations:** 1 Department of Geriatrics, The Affiliated Hospital of Qingdao University, Qingdao, China; 2 Department of Geriatrics, Qingdao Medical College, Qingdao University, Qingdao, China; 3 Senior Department of Nephrology, First Medical Center of Chinese PLA General Hospital, Beijing, China; 4 State Key Laboratory of Kidney Diseases, National Clinical Research Center for Kidney Diseases, Beijing, China; 5 Beijing Key Laboratory of Medical Devices and Integrated Traditional Chinese and Western Drug Development for Severe Kidney Diseases, Beijing, China; 6 Beijing Key Laboratory of Digital Intelligent TCM for the Prevention and Treatment of Pan-vascular Diseases, Key Disciplines of National Administration of Traditional Chinese Medicine (zyyzdxk-2023310), Beijing, China

**Keywords:** anthropometry, arterial stiffness, body mass index, brachial-ankle pulse wave velocity, weight-adjusted waist index

## Abstract

**Background:**

This study aimed to compare the ability of eight commonly used anthropometric indices to identify arterial stiffness (AS) in relatively healthy Chinese adults, providing evidence for early cardiovascular risk screening.

**Methods:**

A total of 1,425 participants were included in this cross-sectional investigation. Eight anthropometric indices [body mass index (BMI), waist-to-hip ratio (WHR), waist-to-height ratio (WHtR), body roundness index (BRI), body adiposity index (BAI), conicity index (CI), body shape index (ABSI), and weight-adjusted waist index (WWI)] were evaluated for their association with brachial-ankle pulse wave velocity (baPWV) using Pearson’s and age-adjusted partial correlations. Logistic regression models were used to evaluate the associations between standardized anthropometric indices and AS. Receiver operating characteristic curves and area under the curve (AUC) were used to assess discriminative ability.

**Results:**

Pearson’s correlation analysis revealed that, except for BMI, the other seven anthropometric indices were significantly positively associated with baPWV, with WWI showing the strongest association, and these associations persisted after adjusting for metabolic syndrome (MetS)-related factors. Notably, WWI exhibited the strongest association with baPWV. Logistic regression analysis showed that, when the variables were not adjusted or only age or MetS components were adjusted, the odds ratios (ORs) for WWI were the largest compared with the other seven indices, at 1.808 (1.542–2.120), 1.282 (1.070–1.537), and 1.839 (1.451–2.330), respectively. Among the eight anthropometric indices, the AUC of WWI was the highest (overall, 0.677; men, 0.628; women, 0.708).

**Conclusion:**

Eight anthropometric indices were positively associated with baPWV, of which WWI had the best predictive ability, providing a basis for the subsequent development of anthropometric indicators.

## 1 Introduction

Arterial stiffness (AS) is increasingly recognized as a key marker of vascular aging and a strong independent predictor of adverse cardiovascular outcomes, including myocardial infarction, stroke, and heart failure (1, 2). AS results from the loss of elastic properties in large arteries, leading to increased pulse wave velocity (PWV) and altered hemodynamics, thereby elevating cardiac load and impairing microvascular function (3). PWV has been repeatedly shown to be a reliable, non-invasive measure with predictive value for cardiovascular risk (4–6).

Anthropometric indices, which estimate overall and central adiposity, are convenient, low-cost tools for evaluating cardiometabolic risk. Body mass index (BMI) is the most commonly used measure for classifying overweight and obesity, but it is affected by age and sex, and it fails to distinguish adipose tissue from muscle mass (7, 8). Waist-to-hip ratio (WHR) and waist-to-height ratio (WHtR) are considered better predictors of metabolic and cardiovascular abnormalities, addressing some limitations of BMI (9–12).

Several novel anthropometric measures have been developed using traditional measurements (height, weight, waist circumference, and hip circumference), including body roundness index (BRI), conicity index (CI), body adiposity index (BAI), a body shape index (ABSI), and weight-adjusted waist index (WWI). These indices have been shown to predict cardiovascular outcomes, metabolic syndrome (MetS), and mortality, with varying strengths across populations (13–18).

Given that MetS components—including central obesity, hypertension, hyperglycemia, and dyslipidemia—are established determinants of AS, it is essential to assess whether anthropometric indices predict AS independently of these factors. Identifying indices with predictive value beyond conventional metabolic risk factors could provide simple, practical tools for early cardiovascular risk screening (19–22).

To our knowledge, few studies have directly compared the associations of these eight anthropometric indices (BMI, WHtR, WHR, BRI, CI, BAI, ABSI, WWI) with brachial-ankle pulse wave velocity (baPWV) in relatively healthy adults. Studies exploring the correlation between anthropometric indicators and AS can help assess the risk of cardiovascular diseases and provide an important reference for the prevention, diagnosis, and treatment of cardiovascular diseases. This may help identify more reliable anthropometric indicators to predict AS and provide a basis for the prevention of AS. Therefore, this study aimed to investigate the relationship between AS (assessed by baPWV) and these anthropometric parameters and to identify the most reliable indices for predicting AS.

## 2 Materials and methods

### 2.1 Study design and participants

This cross-sectional study was conducted in 2016 at the People’s Liberation Army General Hospital, Beijing, China. A total of 2,217 volunteers aged ≥18 years were initially enrolled, of whom 792 were excluded because of (a) respiratory diseases (chronic obstructive pulmonary disease [COPD], asthma, bronchiectasis); (b) musculoskeletal or rheumatic disorders; (c) recent severe diseases (e.g., liver cirrhosis, stroke, myocardial infarction, malignancy); (d) inability to comply with testing; or (e) incomplete data. The final sample comprised 1,425 participants (Supplementary Figure 1). The study protocol was approved by the Ethics Committee of the Chinese People’s Liberation Army General Hospital (No. S2017-133-01) and conducted in accordance with the principles of the Declaration of Helsinki. All participants provided written informed consent prior to participation in the study.

Sociodemographic data, medical and family histories, and laboratory results were collected. Anthropometric measurements were performed by trained personnel following standardized procedures. Participants were measured while wearing light clothing and no shoes. Body height was measured in a standing position, and body weight was measured using a calibrated scale. Waist circumference was measured with a non-elastic measuring tape at the midpoint between the lower rib and the iliac crest at the end of normal expiration, with the participant standing upright. Hip circumference was measured at the level of maximum circumference over the buttocks. The measuring tape was kept horizontal and close to the body without compressing the skin. Blood pressure was measured in the nondominant arm after 5 min of rest and repeated three times. Venous blood samples were collected after a ≥ 12-h fast for measurement of total cholesterol (TC), triglycerides (TG), high-density lipoprotein cholesterol (HDL-C), and other biochemical parameters. The calculation methods for BMI, WHtR, WHR, BRI (23), CI (24), BAI (25), ABSI (26), and WWI (27) are shown in Table 1.

**Table 1:** Calculation formulas of eight anthropometric indicators.

Index	Equation
BMI	BMI = BW _kg_/BH _m_^2^
WHtR	WHtR = WC _cm_/BH _cm_
WHR	WHR = WC _cm_/HC _cm_
BRI	BRI = 364.2–365.5 × [1 − (WC _cm_/2π)^2^/(0.5 * BH _cm_) ^2^]^0.5^
CI	CI=WC _m_/[0.109 * (BW _kg_/BH _m_)^0.5^]
BAI	BAI = HC _cm_/BH _m_^3/2^ −18
ABSI	ABSI=WC _m_/(BMI^2/3^ * BH _m_^1/2^)
WWI	WWI=WC _cm_/BW _kg_^1/2^

WC, waist circumference; HC, hip circumference; BW, body weight; BH, body height; BMI, body mass index; WHR, waist-to-hip ratio; WHtR, waist-to-height ratio; BRI, body roundness index; CI, conicity index; BAI, body adiposity index; ABSI, a body shape index; WWI, weight-adjusted waist index.

### 2.2 Measurements of brachial-ankle pulse wave velocity

Before baPWV assessment, participants were instructed to fast for at least 12 h and to avoid alcohol and vigorous physical activity before the examination. They were also asked to refrain from caffeine-containing beverages and smoking before the measurement. All baPWV measurements were performed in a quiet room after the participants had rested in the supine position for at least 10 min. The baPWV was assessed using a validated automated device (OMRON, BP-203RPEIII, Japan), which records pulse waves in both the brachial and ankle arteries via the oscillometric method while simultaneously measuring blood pressure in the bilateral arms and ankles. Based on prior research, a baPWV value of ≥1,800 cm/s was classified as indicative of AS (28, 29).

### 2.3 Statistical analysis

The normality of continuous variables was evaluated using the Kolmogorov–Smirnov test. Data are presented as the mean ± SD for normally distributed variables and the median (interquartile range) for nonnormally distributed variables. Categorical variables are shown as percentages. Group differences were analyzed with *t*-tests, chi-square tests, or Mann–Whitney U-tests, depending on the data type.

Associations among the eight anthropometric indices, MetS components, and AS were evaluated using Pearson’s correlation, with scatter plots for verification. Partial correlations were calculated after adjustment for age. The magnitude of correlation coefficients was interpreted according to Cohen’s effect-size criteria based on the absolute *r* value: approximately 0.10, small effect size; approximately 0.30, medium effect size; and approximately 0.50, large effect size (30). Restricted cubic spline (RCS) analyses were performed to explore potential nonlinear associations between the eight anthropometric indices and AS. Logistic regression was used to examine associations between anthropometric indices and AS, with baPWV dichotomized at 1,800 cm/s. Multivariable logistic regression adjusting for MetS components (waist circumference [WC], fasting blood glucose [FBG], systolic blood pressure [SBP], diastolic blood pressure [DBP], TG, and HDL-C) was conducted to assess independent associations. Multicollinearity was assessed using the variance inflation factor (VIF), tolerance, condition index, and variance proportions. Results are reported as odds ratios (ORs) with 95% confidence intervals (CIs), using standardized z scores for the indices.

Receiver operating characteristic (ROC) curves and area under the curve (AUC) were used to assess predictive performance, with optimal thresholds determined by the highest Youden index (sensitivity + specificity – 1). The discriminative ability of each anthropometric index was interpreted according to previously published AUC classification criteria: AUC = 0.5, non-informative; 0.5 < AUC ≤ 0.7, less accurate; 0.7 < AUC ≤ 0.9, moderately accurate; 0.9 < AUC < 1.0, highly accurate; and AUC = 1.0, perfect (31). AUC comparisons were performed using the DeLong test. Two-sided *p*-values <0.05 were considered statistically significant.

## 3 Results

### 3.1 Characteristics of participants

A total of 1,425 participants, 602 men and 823 women, were included (Table 2). There were significant differences in age, height, FBG, SBP, DBP, estimated glomerular filtration rate (eGFR), WHR, WHtR, CI, BAI, ABSI, and WWI in men with AS compared with those without AS (*p* < 0.05). There were significant differences in age, height, WC, TG, FBG, SBP, DBP, urea nitrogen, eGFR, and eight anthropometric indices in women with AS compared with those without AS (*p* < 0.05).

**Table 2:** Baseline characteristics of participants according to the degree of arterial stiffness.

	Male(n=602)			Female(n=823)		
Characteristics	baPWV<1800 (cm/s) (n=517)	baPWV≥1800 (cm/s) (n=85)	P-value	baPWV<1800 (cm/s) (n=698)	baPWV≥1800 (cm/s) (n=125)	P-value
Age (years)	56.14±13.25	69.15±9.428	<0.001	55.84±12.516	71.05±8.888	<0.001
Height (cm)	171.58±5.594	169.33±5.458	0.001	159.90±5.105	157.81±4.758	<0.001
Weight (kg)	73.248±11.672	70.941±9.735	0.085	61.834±9.256	60.336±8.958	0.094
WC (cm)	91.22±9.265	92.08±8.162	0.42	82.88±9.256	86.57±9.285	<0.001
HC (cm)	100.05±5.929	99.56±5.231	0.474	97.94±6.638	98.53±7.665	0.426
BMI (kg/m2)	24.833±3.465	24.707±2.933	0.752	24.185±3.473	24.184±3.097	0.998
TG (mmol/L)	1.725±1.397	1.761±1.31	0.822	1.416±0.916	1.604±0.847	0.033
TC (mmol/L)	4.61(3.98,5.2)	4.52(3.87,5.3)	0.7	4.79(4.2,5.472)	4.89(4.18,5.39)	0.894
HDL-C (mmol/L)	1.31±0.34	1.312±0.328	0.963	1.541±0.383	1.514±0.37	0.46
SBP (mmHg)	124.17±13.451	141.07±13.95	<0.001	121.1±15.42	141.12±15.538	<0.001
DBP (mmHg)	74.58±9.407	81.35±8.811	<0.001	69.98±9.702	76.48±9.37	<0.001
FBG (mmol/L)	5.492±1.497	5.94±2.465	0.022	5.287±1.185	6.222±1.863	<0.001

BUN (mmol/L)	5.393±1.395	5.666±1.043	0.086	4.816±1.27	5.33±1.226	<0.001
eGFR (mL/min/1.73 m2)	93.565±19.002	80.899±14.876	<0.001	91.163±16.99	79.066±13.823	<0.001
WHR	0.91±0.058	0.923±0.051	0.046	0.845±0.062	0.879±0.065	<0.001
WHtR	0.532(0.496,0.566)	0.538(0.518,0.570)	0.046	0.518(0.475,0.560)	0.544(0.506,0.584)	<0.001
BRI	4.028±1.052	4.263±0.98	0.055	3.785±1.18	4.38±1.195	<0.001
CI	1.285±0.087	1.307±0.061	0.022	1.225±0.095	1.287±0.086	<0.001
BAI	26.562±2.789	27.237±2.678	0.038	30.505±3.672	31.739±3.934	0.001
ABSI	0.082±0.006	0.084±0.004	0.026	0.079±0.006	0.083±0.005	<0.001
WWI	10.694±0.748	10.954±0.527	0.002	10.566±0.846	11.167±0.761	<0.001

Categorical variables are presented as the percentages, and continuous variables are described as the means ± standard deviation (SD) for normally distributed data or the medians (interquartile range) for skewed data. WC, Waist circumference; HC, Hip circumference; BMI, Body mass index; FBG, Fasting blood glucose; TG, Triglycerides; TC, Total cholesterol; HDL-C, High-density lipoprotein cholesterol; SBP, Systolic blood pressure; DBP, Diastolic blood pressure; BUN, blood urea nitrogen; eGFR, estimated glomerular filtration rate; WHR, waist-to-hip ratio; WHtR, waist-to-height ratio; BRI, Body roundness index; CI, conicity index; BAI, Body adiposity index; ABSI, a body shape index; WWI, weight-adjusted-waist index.

### 3.2 Changes in baPWV across quartiles of the eight anthropometric indicators

**Figure 1:**
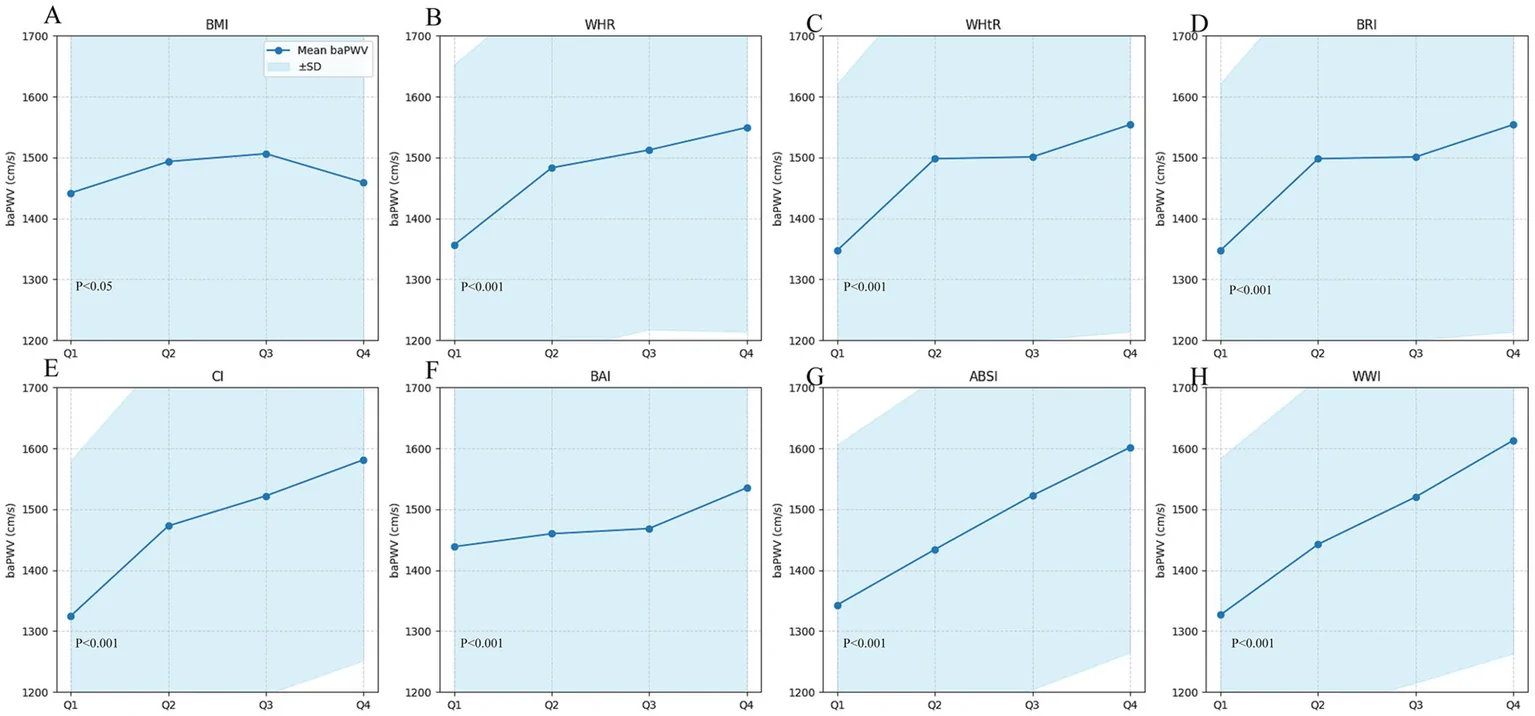
Change in baPWV across quartiles of the eight anthropometric measures. **(A)** BMI, **(B)** WHR, **(C)** WHtR, **(D)** BRI, **(E)** CI, **(F)** BAI, **(G)** ABSI, and **(H)** WWI.

We depicted the change in baPWV across quartiles of the eight anthropometric indicators (Figure 1). The mean baPWV increased significantly across quartiles of BMI, WHtR, WHR, BRI, CI, BAI, ABSI, and WWI, with WWI values as follows: (Group I [lowest]: 1,326.88 ± 257.83 vs. Group II: 1,443.50 ± 272.88 vs. Group III: 1,517.22 ± 304.10 vs. Group IV [highest]: 1,613.23 ± 349.73 cm/s; *p* < 0.01).

### 3.3 Association between baPWV and eight anthropometric indices

**Figure 2:**
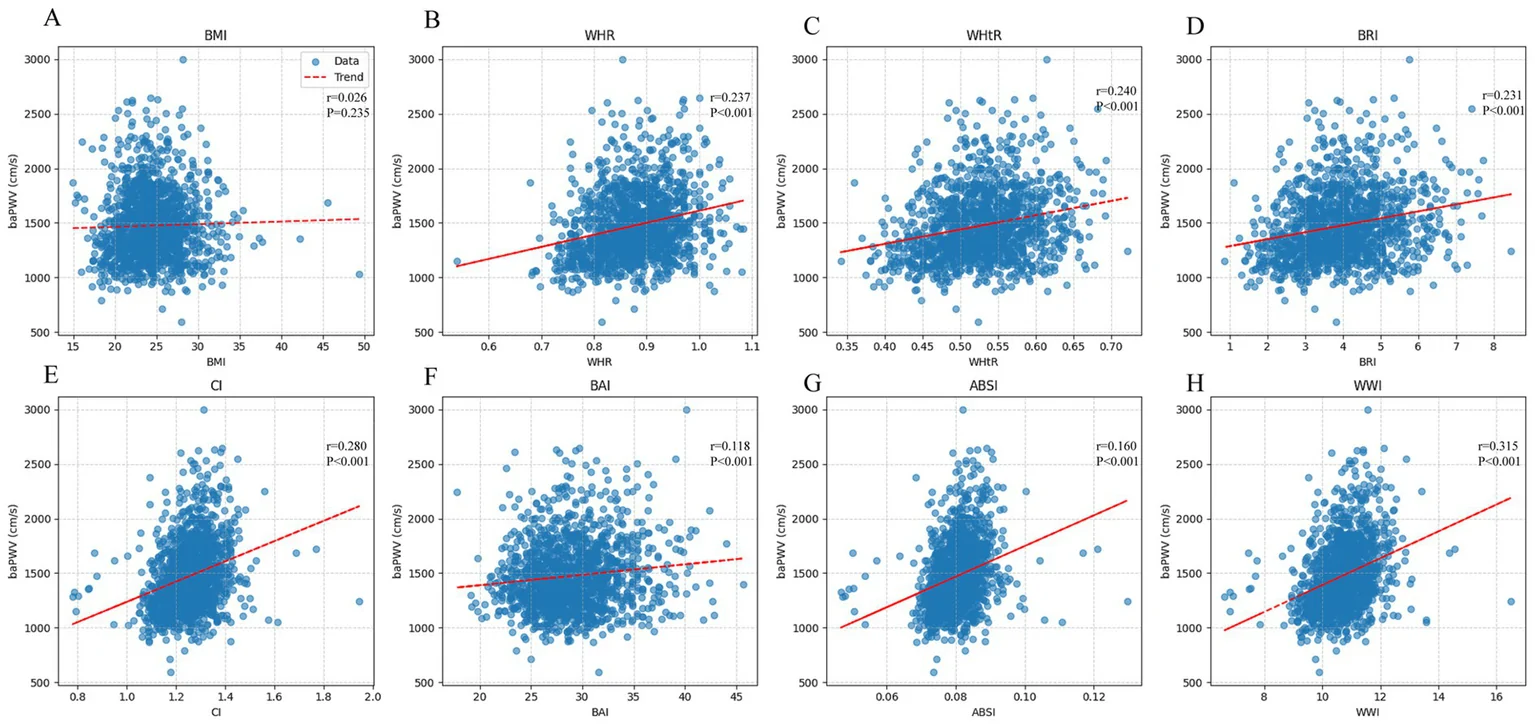
Associations between baPWV and eight anthropometric indices. **(A)** BMI, **(B)** WHR, **(C)** WHtR, **(D)** BRI, **(E)** CI, **(F)** BAI, **(G)** ABSI, and **(H)** WWI.

We described the associations between baPWV and eight anthropometric indicators and components of MetS (Figure 2; Supplementary Table 1). Pearson’s correlation analysis showed that, except for BAI, the other seven indices were correlated with MetS components. At the same time, except for BMI, the remaining seven anthropometric indices were significantly positively correlated with baPWV (*p* < 0.001); among these indices, WWI showed the strongest correlation with baPWV. According to Cohen’s effect-size criteria, the magnitude of this correlation was classified as a medium effect-size correlation, although the value was modest and only slightly above the threshold for a medium effect size (*r* = 0.315, *p* < 0.001). After adjusting for age, partial correlation analysis showed that, except for BAI, the other seven indices were correlated with MetS components; meanwhile, except for BMI, the remaining seven anthropometric indices were significantly positively correlated with baPWV (*p* < 0.05). However, the magnitude of this association decreased and was classified as a small effect-size correlation according to Cohen’s criteria (*r* = 0.147, *p* < 0.001). In addition, baPWV was significantly associated with MetS components regardless of age adjustment, with the strongest association with SBP.

### 3.4 Logistic regression analysis of the predictive value of eight anthropometric indices for AS

In the univariable logistic regression analysis, in the unadjusted model, WWI had the largest OR value of 1.808 (95% CI 1.542–2.120, *p* < 0.001). In the multivariable logistic regression analysis, after adjusting for age, WWI still had the largest OR value of 1.282 (95% CI 1.070–1.537, *p* = 0.007). After adjusting for MetS components (WC, FBG, SBP, DBP, TG, and HDL-C), the OR value of CI was 1.912 (95% CI 1.448–2.526, *p* < 0.001), followed by the OR value of WWI, which was 1.839 (95% CI 1.451–2.330, *p* < 0.001), indicating that CI and WWI predicted AS independently of conventional metabolic risk factors (Table 3).

**Table 3:** Multivariate logistic regression analysis of the predictive value of eight anthropometric indices for AS.

Variable	Model I OR (95% CI)	*P* value	Model II OR (95% CI)	*P* value	Model III OR (95% CI)	*P* value
BMI	0.981 (0.847–1.137)	0.799	1.107 (0.935–1.311)	0.239	0.610 (0.467–0.796)	<0.001
WHtR	1.488 (1.281–1.728)	<0.001	1.260 (1.062–1.495)	0.008	1.669 (1.018–2.513)	0.014
WHR	1.434 (1.233–1.669)	<0.001	1.217 (1.020–1.453)	0.03	1.683 (1.238–2.288)	0.001
BRI	1.458 (1.263–1.683)	<0.001	1.241 (1.052–1.463)	0.01	1.536 (1.041–2.266)	0.030
CI	1.626 (1.389–1.903)	<0.001	1.233 (1.025–1.483)	0.026	1.912 (1.448–2.526)	<0.001
BAI	1.307 (1.134–1.505)	<0.001	1.156 (0.991–1.348)	0.66	1.031 (0.865–1.230)	0.732
ABSI	1.606 (1.374–1.878)	<0.001	1.182 (0.981–1.423)	0.078	1.572 (1.276–1.936)	<0.001
WWI	1.808 (1.542–2.120)	<0.001	1.282 (1.070–1.537)	0.007	1.839 (1.451–2.330)	<0.001

BMI, body mass index; WHR, waist-to-hip ratio; WHtR, waist-to-height ratio; BRI, body roundness index; CI, conicity index; BAI, body adiposity index; ABSI, a body shape index; WWI, weight-adjusted waist index. Model I, unadjusted; Model II, adjusted for age; Model III, adjusted for MetS.

### 3.5 Comparison of the ability of eight anthropometric indices to identify AS

**Figure 3:**
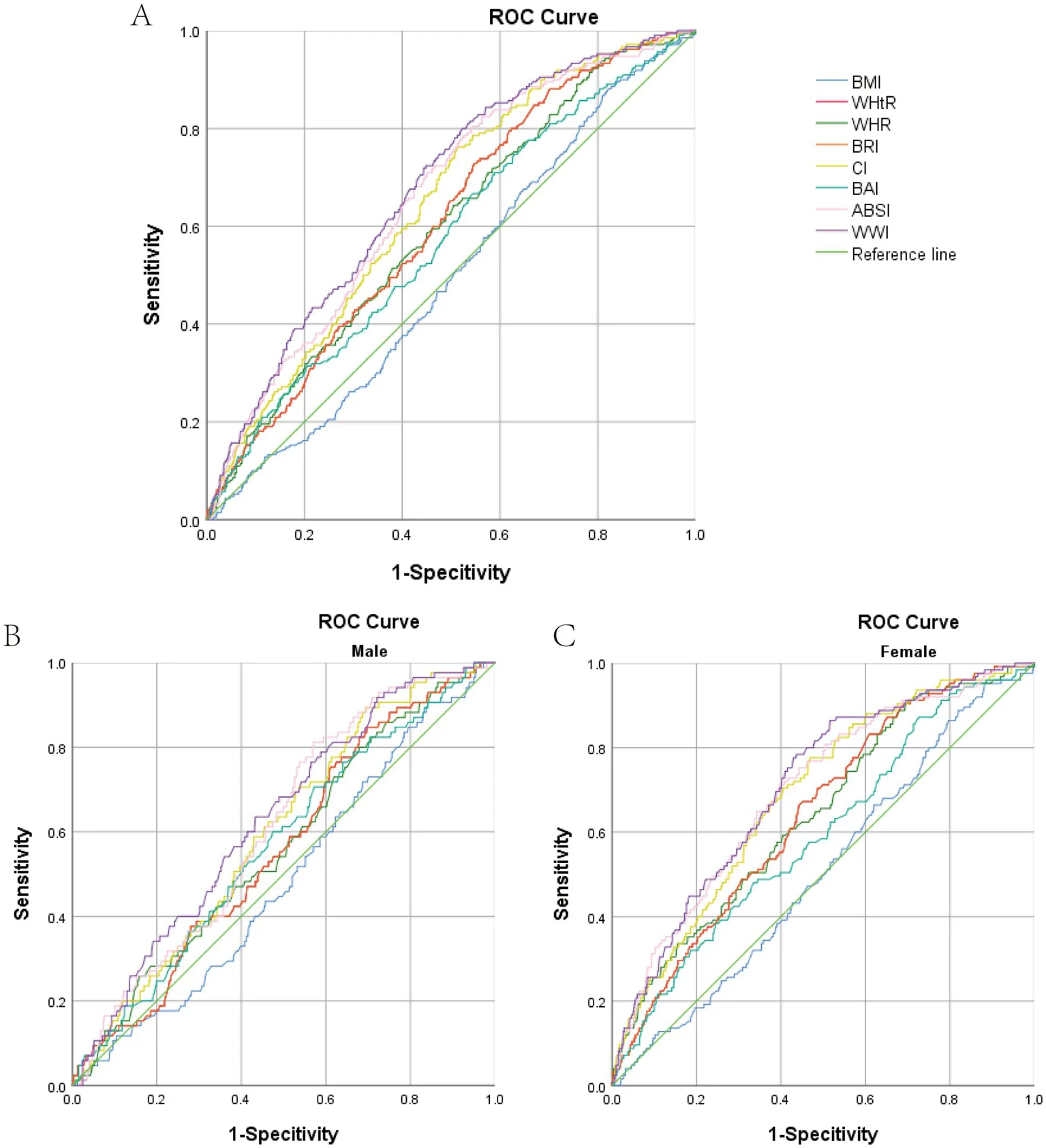
Comparison of the diagnostic values of BMI, WHtR, WHR, BRI, CI, BAI, ABSI, and WWI in predicting AS in a relatively healthy Chinese population. **(A)** Both sex groups; **(B)** Male; **(C)** Female.

We analyzed the AUC values of each anthropometric measure for predicting AS, both men and women, and BMI had the lowest AUC in identifying AS (overall, 0.498; male, 0.486; female, 0.508). WWI had the highest AUC among the eight anthropometric indices (overall, 0.677; male, 0.628; female, 0.708) (Figure 3; Table 4). According to the predefined AUC classification criteria, WWI showed less accurate discriminative ability in the overall population and in men, and moderately accurate discriminative ability in women. The ROC curves of the eight indices are shown in Supplementary Figure 2.

**Table 4:** Area under the curve of eight anthropometric indices for predicting AS.

Variable	Overall		*P* value	Men		*P* value	Women		*P* value
	AUC	95% CI		AUC	95% CI		AUC	95% CI	
BMI	0.498	0.458–0.539	0.939	0.486	0.424–0.549	0.688	0.508	0.455–0.56	0.785
WHtR	0.609	0.57–0.647	<0.001	0.56	0.498–0.621	0.078	0.643	0.594–0.692	<0.001
WHR	0.599	0.559–0.639	<0.001	0.563	0.5–0.626	0.063	0.642	0.591–0.693	<0.001
BRI	0.609	0.57–0.647	<0.001	0.56	0.498–0.621	0.078	0.643	0.594–0.692	<0.001
CI	0.646	0.608–0.683	<0.001	0.598	0.539–0.657	0.004	0.688	0.64–0.736	<0.001
BAI	0.576	0.534–0.617	<0.001	0.568	0.505–0.631	0.044	0.593	0.539–0.646	0.001
ABSI	0.660	0.622–0.698	<0.001	0.613	0.555–0.671	0.001	0.698	0.649–0.747	<0.001
WWI	0.677	0.641–0.714	<0.001	0.628	0.569–0.687	<0.001	0.708	0.661–0.755	<0.001

BMI, body mass index; WHR, waist-to-hip ratio; WHtR, waist-to-height ratio; BRI, body roundness index; CI, conicity index; BAI, body adiposity index; ABSI, a body shape index; WWI, weight-adjusted waist index.

According to the DeLong test, we compared the AUC values of eight indices (Supplementary Table 2), and WWI differed significantly from the other six indices (BMI, WHtR, WHR, BRI, CI, BAI) in identifying AS (*p <* 0.05). The difference between ABSI and WWI did not reach the traditional statistical significance level (*p =* 0.086). We calculated the best cutoff values for each index to assess the prediction ability of AS. The best cutoff value for WWI was 10.55 (sensitivity 80%, specificity 47.7%), that for men was 10.75 (sensitivity 63.5%, specificity 56.7%), and that for women was 10.50 (sensitivity 86.4%, specificity 48.4%) (Supplementary Table 3). RCS analyses were performed to further explore potential nonlinear associations between the eight anthropometric indices and AS. No significant nonlinear associations were observed for BMI, WHR, WHtR, BRI, CI, BAI, ABSI, or WWI (all *P* for nonlinearity >0.05; Supplementary Figure 2).

## 4 Discussion

This study contributes new insights to existing research on the relationship between anthropometric indices and AS by specifically comparing the performance of eight commonly used indicators, including BMI, WHtR, WHR, BRI, CI, BAI, ABSI, and WWI, in identifying AS within a relatively healthy Chinese adult population. We identified positive associations between baPWV and most anthropometric indices, suggesting that obesity-related body size and fat distribution may be associated with arterial stiffness risk even in individuals without severe underlying diseases. Notably, WWI demonstrated the strongest association with baPWV and the highest discriminative ability among the eight indices evaluated. According to Cohen’s effect-size criteria, the unadjusted correlation between WWI and baPWV was modest-to-moderate in magnitude, whereas the age-adjusted correlation was small.

BMI is the most commonly used anthropometric measure, but its use as a measure of AS is still controversial. In our study, there was no significant correlation between BMI and baPWV, which is consistent with the results of a recent study of 3,758 healthy subjects. Kim et al. showed that baPWV was associated with WHR, but not BMI (32). This means that AS may be more closely associated with abdominal obesity than with overall obesity. Zuo et al. investigated 857 adults and reported that a linear association between BMI and baPWV was observed only in men under 50 years (*p* = 0.01) and women aged 50 years or older (*p* < 0.01), suggesting that BMI may not serve as an optimal indicator of obesity for evaluating cardiovascular risk (33). These studies suggest a weak correlation between AS and overall obesity.

WHtR, WHR, and BRI are commonly used to evaluate MetS (34, 35). WHtR, WHR, and BRI are among the more common anthropometric indices. In a meta-analysis, WHtR, WHR, and BRI were found to be closely related to cardiovascular risk (36). In a cohort of 658 healthy Persians, WHtR, WHR, and BRI were significantly associated with AS (as defined by carotid-femoral PWV), and improving these indices may help prevent cardiovascular disease (CVD) (37). In this study, we found that the AUC values of WHtR and BRI were the same in predicting AS, which may be because both WHtR and BRI were calculated using height and WC.

In addition, Haraguchi et al. used BMI, ABSI, and BRI to evaluate AS. ABSI was found to predict AS with high accuracy by assessing fat distribution in non-obese men. At the same time, ABSI and BRI may be used as predictors of vascular remodeling or organic vascular dysfunction (38). Li et al. analyzed 11,872 patients with type 2 diabetes and found that combining ABSI with BMI provided a clearer understanding of mortality risk variations than using BMI or other measures alone (39). Kajikaw et al. investigated how ABSI relates to endothelial function in a cohort of 8,823 participants, including 6,773 men and 2,050 women. Their findings indicated that ABSI independently predicts endothelial dysfunction across genders and can be used to evaluate cardiovascular risk (40). In a study of 62,514 Japanese participants, ABSI was found to be an easy-to-calculate indicator of abdominal obesity, reflecting metabolic disorders and systemic AS, which can be used for primary health screening (41). In a study using healthy Chinese, hypertensive, and diabetic participants, WHtR, ABSI, and BRI were found to be better predictors of AS in both men and women (42). In our study, we also found that WHtR, ABSI, and BRI were significantly correlated with baPWV.

A Brazilian study on 706 participants found that BAI has some capacity to forecast body fat (43). In a study of 2,425 children and 5,726 adults in rural China, BMI was found to be a more suitable proxy for body fat than BAI (44). In our study, the correlation between BAI and baPWV was small. After adjusting for age variables, the association was not statistically significant. BAI as a surrogate index of body fat and its relationship with AS need further study. In 1993, Valdez et al. proposed a simple anthropometric index, CI, to assess obesity and body fat distribution (45). The study suggested that central obesity was more strongly associated with CVD than systemic obesity (24, 46, 47). Nkwana et al. examined 624 rural South African youths and reported associations of BRI, ABSI, and CI with nutritional status and cardiovascular risk factors, finding that CI was significantly associated with insulin resistance, hypertension, and dyslipidemia (48). CI was found to be better at predicting MetS among women in a study of 160 apparently healthy adults with normal blood glucose and blood pressure in rural China (49). Currently, no studies have explored the link between CI and AS among Chinese adults. Our study found that CI was significantly correlated with MetS components and baPWV and showed certain predictive ability for AS.

In 2018, Park et al. introduced WWI as a straightforward anthropometric indicator for obesity by normalizing waist circumference with body weight. Analysis of 465,629 individuals from the Korean National Health Insurance Cohort revealed that WWI outperformed BMI, WC, WHtR, and ABSI in predicting cardiovascular mortality (27). In a study involving 602 adults aged 65 years and older, WWI was identified as an anthropometric measure positively associated with fat mass and inversely related to muscle mass, highlighting its utility in evaluating body composition (50). Furthermore, Zhu et al. found that higher WWI levels were strongly associated with increased risks of prehypertension and hypertension, and incorporating WWI into conventional models improved the prediction of these conditions (51). Among 5,232 hypertensive patients, WWI was closely linked to AS, suggesting that WWI could serve as a target for AS prevention and management alongside conventional blood pressure control (52). A study in northern Chinese adults reported that novel obesity indicators, especially WWI, outperformed traditional obesity measures in predicting AS (53). In another 2026 study of a Middle Eastern population, WWI and the visceral adiposity index (VAI) showed significant associations with AS in coronary artery disease patients (54). In the present study, WWI demonstrated significant associations with MetS components and baPWV. After adjustment for potential confounders, WWI showed stronger correlations with baPWV than other anthropometric indices and exhibited the highest AUC for identifying AS. However, according to the predefined AUC classification criteria, the discriminative ability of WWI was less accurate in the overall population and in men and moderately accurate in women. These findings suggest that WWI may be useful as a simple preliminary screening marker for AS, but it should not be considered a standalone diagnostic tool. Although the difference in AUC between WWI and ABSI for predicting AS was not statistically significant, given that both indices are calculated from similar anthropometric measures and that WWI is simpler to compute, we propose that WWI may serve as a superior anthropometric indicator for assessing AS compared with the other seven indices. Regular monitoring of this straightforward, non-invasive index, combined with timely interventions such as physical activity and dietary improvements, may help mitigate AS and its associated complications. These findings provide a reference framework for the clinical use of WWI to assess AS.

The study has several limitations. First, the cross-sectional design and relatively small sample size of this study preclude any conclusions regarding causal relationships. Second, the study population consisted predominantly of relatively healthy individuals from China, which may limit the generalizability of the findings to other ethnicities or populations with different health profiles. Third, although WWI showed the strongest correlation with baPWV and the highest AUC among the eight anthropometric indices, the magnitude of these associations should be interpreted cautiously. According to Cohen’s effect-size criteria, the unadjusted correlation between WWI and baPWV was modest-to-moderate, whereas the age-adjusted correlation was small. In addition, the overall discriminative ability of WWI remained limited. Therefore, WWI may be more appropriate as a simple preliminary risk-screening marker rather than a standalone diagnostic indicator of AS. Fourth, although RCS analyses were performed, no significant nonlinear associations were observed between the eight anthropometric indices and AS. This may be partly related to the relatively healthy study population and the limited discriminatory ability of single anthropometric markers. Future studies should evaluate whether combining WWI with clinical variables and biochemical markers can improve the prediction of AS.

## 5 Conclusion

Our results showed that WWI was significantly associated with AS and positively correlated with MetS components, except HDL-C. Among the eight anthropometric indices, WWI showed the highest, although limited, discriminative ability for identifying AS. These findings suggest that WWI may serve as a simple and practical anthropometric marker for preliminary AS risk screening in relatively healthy Chinese adults.

## Data Availability

The raw data supporting the conclusions of this article will be made available by the authors, without undue reservation.
