# Supplementary material for: Correlation analysis between eight anthropometric indicators and arterial stiffness in relatively healthy Chinese adults
Source: Front Nutr. 2026 Aug 17;13:1873108. doi: 10.3389/fnut.2026.1873108 (PMC13526973; doi:10.3389/fnut.2026.1873108)
Supplement: Supplementary file 1 [file Supplementary_file_1.DOCX]

Supplementary Material

# 1 Supplementary Data

# Supplementary Table 1: Association between baPWV and eight anthropometric indices and components of metabolic syndrome.

|  |  | WC | FBG | SBP | DBP | TG | HDL-C | baPWV |
| --- | --- | --- | --- | --- | --- | --- | --- | --- |
| Unadjusted | BMI | 0.68a | 0.119a | 0.184a | 0.196a | 0.21a | -0.327a | 0.026  P=0.325 |
|  | WHtR | 0.908a | 0.219a | 0.327a | 0.271a | 0.232a | -0.342a | 0.237a |
|  | WHR | 0.84a | 0.219a | 0.275a | 0.311a | 0.249a | -0.366a | 0.240a |
|  | BRI | 0.902a | 0.219a | 0.32a | 0.262a | 0.227a | -0.331a | 0.231a |
|  | CI | 0.78a | 0.195a | 0.287a | 0.251a | 0.177a | -0.276a | 0.280a |
|  | BAI | 0.298a | 0.082  P=0.002 | 0.178a | 0.023  P=0.39 | 0.036  P=0.18 | -0.025  P=0.349 | 0.118a |
|  | ABSI | 0.57a | 0.157a | 0.23a | 0.189a | 0.112a | -0.173a | 0.271a |
|  | WWI | 0.668a | 0.198a | 0.295a | 0.203a | 0.139a | -0.184a | 0.315a |
|  | baPWV | 0.176a | 0.259a | 0.658a | 0.448a | 0.098a | -0.014a | 1 |
| Adjusted | BMI | 0.69a | 0.122a | 0.202a | 0.2a | 0.21a | -0.327a | 0.04  P=0.135 |
|  | WHtR | 0.908a | 0.185a | 0.263a | 0.248a | 0.246a | -0.378a | 0.126a |
|  | WHR | 0.837a | 0.186a | 0.207a | 0.29a | 0.263a | -0.403a | 0.13a |
|  | BRI | 0.901a | 0.187a | 0.258a | 0.239a | 0.24a | -0.366a | 0.122a |
|  | CI | 0.78a | 0.152a | 0.199a | 0.223a | 0.194a | -0.322a | 0.14a |
|  | BAI | 0.282a | 0.058  P=0.029 | 0.135a | 0.003  P=0.905 | 0.04  P=0.135 | -0.039  P=0.136 | 0.045  P=0.093 |
|  | ABSI | 0.557a | 0.111a | 0.188a | 0.168a | 0.126a | -0.214a | 0.126a |
|  | WWI | 0.666a | 0.148a | 0.188a | 0.168a | 0.159a | -0.236a | 0.147a |
|  | baPWV | 0.111a | 0.2a | 0.589a | 0.453a | 0.135a | -0.081a | 1 |

BMI, Body mass index; WHR, waist-to-hip ratio; WHtR, waist-to-height ratio; BRI, Body roundness index; CI, conicity index; BAI, Body adiposity index; ABSI, a body shape index; WWI, weight-adjusted-waist index; ^a^:*P* < 0.001.

# Supplementary Table 2: Pairwise comparisons of the AUCs of eight anthropometric indices in predicting atherosclerosis by the Delong test.

|  | BMI | WHtR | WHR | BRI | CI | BAI | ABSI |
| --- | --- | --- | --- | --- | --- | --- | --- |
| WHtR | 2.845  P =0.0044 |  |  |  |  |  |  |
| WHR | 2.862  P = 0.0042 | 0.611  P = 0.5409 |  |  |  |  |  |
| BRI | 2.845  P = 0.0044 | 0  P = 1.000 | 0.611  P = 0.5409 |  |  |  |  |
| CI | 4.551  P < 0.0001 | 2.688  P = 0.0072 | 3.851  P = 0.0001 | 2.688  P = 0.0072 |  |  |  |
| BAI | 2.052  P = 0.0402 | 1.729  P = 0.0837 | 0.726  P = 0.4678 | 1.729  P = 0.0837 | 2.61  P = 0.0091 |  |  |
| ABSI | 5.773  P < 0.0001 | 2.642  P = 0.0082 | 4.025  P = 0.0001 | 2.642  P = 0.0082 | 2.077  P = 0.0378 | 2.879  P =0.0040 |  |
| WWI | 5.716  P < 0.0001 | 5.209  P < 0.0001 | 4.817  P< 0.0001 | 5.209  P < 0.0001 | 3.958  P = 0.0001 | 4.524  P < 0.0001 | 1.717  P = 0.0859 |

BMI, Body mass index; WHR, waist-to-hip ratio; WHtR, waist-to-height ratio; BRI, Body roundness index; CI, conicity index; BAI, Body adiposity index; ABSI, a body shape index; WWI, weight-adjusted-waist index.

# Supplementary Table 3: Optimal cut-off values of 8 indices for predicting AS.

|  | Optimal Cut-Offs | Sensitivity (%) | Specificity (%) | Youden Index |
| --- | --- | --- | --- | --- |
| ALL |  |  |  |  |
| BMI | 21.17 | 89.5 | 16.5 | 0.06 |
| WHtR | 0.52 | 72.9 | 45.2 | 0.181 |
| WHR | 0.87 | 64.3 | 49.5 | 0.138 |
| BRI | 3.70 | 72.9 | 45.2 | 0.181 |
| CI | 1.25 | 76.2 | 48.8 | 0.25 |
| BAI | 28.23 | 66.7 | 45.6 | 0.123 |
| ABSI | 0.079 | 71.2 | 60.3 | 0.315 |
| WWI | 10.55 | 80 | 47.7 | 0.227 |
| Male |  |  |  |  |
| BMI | 21.30 | 90.6 | 15.9 | 0.065 |
| WHtR | 0.51 | 84.7 | 30.4 | 0.151 |
| WHR | 0.89 | 80 | 32.1 | 0.121 |
| BRI | 3.48 | 84.7 | 69.6 | 0.151 |
| CI | 1.25 | 89.4 | 30.6 | 0.2 |
| BAI | 25.88 | 70.6 | 42.7 | 0.133 |
| ABSI | 0.081 | 81.2 | 42.9 | 0.241 |
| WWI | 10.75 | 63.5 | 56.7 | 0.202 |
| Female |  |  |  |  |
| BMI | 20.33 | 95.2 | 11.7 | 0.069 |
| WHtR | 0.50 | 83.2 | 39.1 | 0.223 |
| WHR | 0.81 | 91.2 | 29.8 | 0.21 |
| BRI | 3.33 | 83.2 | 39.1 | 0.223 |
| CI | 1.23 | 77.6 | 53.2 | 0.308 |
| BAI | 28.31 | 87.2 | 27.4 | 0.146 |
| ABSI | 0.081 | 81.2 | 42.0 | 0.241 |
| WWI | 10.50 | 86.4 | 48.4 | 0.348 |

BMI, Body mass index; WHR, waist-to-hip ratio; WHtR, waist-to-height ratio; BRI, Body roundness index; CI, conicity index; BAI, Body adiposity index; ABSI, a body shape index; WWI, weight-adjusted-waist index.

## 1.1 Supplementary Figures

**Supplementary Figure 1.** The flow diagram shows the screening and enrollment of the participants.

**Supplementary Figure 2.** Restricted cubic spline analyses of the associations between the eight anthropometric indices and arterial stiffness. The solid line represents the odds ratio for arterial stiffness, and the shaded area represents the 95% confidence interval. The horizontal dashed line indicates an odds ratio of 1. AS, arterial stiffness; BMI, body mass index; WHR, waist-to-hip ratio; WHtR, waist-to-height ratio; BRI, body roundness index; CI, conicity index; BAI, body adiposity index; ABSI, a body shape index; WWI, weight-adjusted waist index.
